# Performance Analysis and Optimization of a High-Temperature PEMFC Vehicle Based on Particle Swarm Optimization Algorithm

**DOI:** 10.3390/membranes11090691

**Published:** 2021-09-07

**Authors:** Yanju Li, Zheshu Ma, Meng Zheng, Dongxu Li, Zhanghao Lu, Bing Xu

**Affiliations:** College of Automobile and Traffic Engineering, Nanjing Forestry University, Nanjing 210037, China; liyanju@njfu.edu.cn (Y.L.); zhengmeng@njfu.edu.cn (M.Z.); lidongxu@njfu.edu.cn (D.L.); luzhanghao@njfu.edu.cn (Z.L.); xubing@njfu.edu.cn (B.X.)

**Keywords:** HT-PEMFC, parametric studies, particle swarm optimization, powertrain design, simulation analysis

## Abstract

In this paper, a high-temperature proton exchange membrane fuel cell (HT-PEMFC) model using the polybenzimidazole membrane doped with phosphoric acid molecules is developed based on finite time thermodynamics, considering various polarization losses and losses caused by leakage current. The mathematical expressions of the output power density and efficiency of the HT-PEMFC are deduced. The reliability of the model is verified by the experimental data. The effects of operating parameters and design parameters on the output performance of the HT-PEMFC are further analyzed. The particle swarm optimization (PSO) algorithm is used for the multi-objective optimization of the power density and efficiency of the HT-PEMFC. The results show that the output performance of the optimized HT-PEMFC is improved. Then, according to the different output performance of the low-temperature proton exchange membrane fuel cell (LT-PEMFC), HT-PEMFC, and optimized HT-PEMFC, different design schemes are provided for a fuel cell vehicle (FCV) powertrain. Simulation tests are conducted under different driving cycles, and the results show that the FCV with the optimized HT-PEMFC is more efficient and consumes less hydrogen.

## 1. Introduction

With the environmental degradation and energy decay caused by conventional internal combustion engines, new/sustainable energy development is essential [1,2,3,4,5,6,7,8]. In particular, the PEMFC is widely used in FCVs due to its very high energy efficiency and emissions of only water, electricity, and heat [9,10]. Most of the existing FCVs use LT-PEMFCs, but researchers show that the PEMFC requires higher operating temperature conditions to improve its catalyst reaction kinetics [11]. Compared with the LT-PEMFC, the HT-PEMFC [12,13] simplifies the internal water and heat management and improves the CO tolerance of the proton exchange membrane [14,15,16].

At present, the research on HT-PEMFCs mainly includes membrane material [17,18,19,20,21,22], structure design [23], parameter study [24], and performance optimization [25]. Yang et al. [26,27] studied the high-temperature proton exchange membrane (PEM) in order to improve the performance of the PEMFC at high temperature. The results show that the improved membrane based on Nafion cannot guarantee the comprehensive performance under high temperature and low humidity conditions. However, phosphoric-acid-doped polybenzimidazole (PA/PBI) membranes [18] could maintain good mechanical properties and excellent proton conductivity at high temperature and low humidity. In fact, polybenzimidazole (PBI) is an amorphous rigid polymer that is usually doped with phosphoric acid. Phosphoric acid is a good electrolyte with low vapor pressure and high thermal stability at high temperatures compared to other acids [28]. As a result, PBI membranes have good chemical resistance and excellent mechanical strength at high temperatures. Li et al. [29] studied the CO tolerance of HT-PEMFCs based on PBI membranes at high temperature. The experimental results show that with the increase in operating temperature, the CO tolerance of the film is higher, and the performance output is better. Khan [30] et al. proposed a semi-empirical model of HT-PEMFCs considering hydrogen pressure, ambient temperature, pressure, and load resistance. The effect of these parameters on the cell performance was investigated, and the results showed that the output voltage of the HT-PEMFC decreases when the ambient temperature increases and the pressure decreases. Li et al. [31] established an irreversible model of the PEMFC which took leakage current into account and studied the influence of operating temperature and operating pressure. In this paper, the mathematical model of the HT-PEMFC was established based on a PBI membrane, and its performance was analyzed and optimized.

The purpose of applying finite-time thermodynamic theory is to find out the optimal thermodynamic performance of the HT-PEMFC in finite time or finite size to improve the actual output performance [32,33,34,35,36]. Li et al. [37,38,39] developed a mathematical model of the PEMFC based on finite-time thermodynamics. The optimization of its operating and design parameters leads to the reduction of irreversible losses and the improvement of the actual output performance. Lu et al. [40] conducted a fire use analysis of the HT-PEMFC power generation system and established a mathematical model. A new design of Farmland Fertility Optimization (FFO) for optimizing the parameters was proposed, which has the best optimization effect compared with the original design method and genetic algorithm (GA). Li et al. [41] analyzed the PEMFC impact parameters and applied GA to propose the optimal parameter design scheme. The experimental results show that the performance of the PEMFC is improved by 35.8%.

Based on the above research, this paper focuses on the performance analysis and parameter optimization of the HT-PEMFC. The output performance of different PEMFCs provides different design schemes for FCV, and then through simulation tests, we study the performance of the optimized HT-PEMFC on the vehicle. The rest of the paper is organized as follows: Section 2 establishes the HT-PEMFC model for validation with experimental data; Section 3 and Section 4 analyze and optimize the parameters of the HT-PEMFC; Section 5 provides powertrain design solutions for FCV based on the different output performance of the PEMFC; finally, conclusions are given in Section 6.

## 2. HT-PEMFC Model

A single PEMFC mainly consists of a bipolar plate, a gas diffusion layer (GDL), a catalytic layer (GL), and a PEM [42]. The power density and efficiency of the PEMFC have great influence on the performance of the FCV. The electrochemical reactions at the cathode and anode of the HT-PEMFC are $2H^{+}+\frac{1}{2}O_{2}+2e^{-}\to H_{2}O$ and $H_{2}\to 2H^{+}+2e^{-}$, respectively. The total reaction is $H_{2}\left(g\right)+\frac{1}{2}O_{2}\left(g\right)\to H_{2}O\left(g\right)+\mathrm{heat}+\mathrm{electricity}$. By analyzing the internal reaction process of the HT-PEMFC, the mathematical model of a single HT-PEMFC was established based on electrochemistry and thermodynamics [43].

### 2.1. Reversible Output Voltage

The concentration and operating pressure of the reaction gas affect the Gibbs free energy. For the HT-PEMFC, the reversible output voltage $E_{r}$ is as follows [44]:

(1) $$E_{r}=E_{r}^{0}+\frac{\Delta S}{nF}\left(T-T_{0}\right)+\frac{RT}{nF}ln(\frac{p_{H_{2}}p_{O_{2}}^{0.5}}{p_{H_{2}O}})$$

where $E_{r}^{0}$ is the ideal standard potential, which is 1.185 V [45]; n is the number of electrons exchanged per hydrogen molecule; F is the Faraday constant; T is the operating temperature; ΔS is the standard molar enthalpy; R is a gas constant; and $p_{H_{2}}$, $p_{O_{2}}$, and $p_{H_{2}O}$ are partial pressures of hydrogen, oxygen, and water vapor, respectively. ΔS is the change of standard molar entropy, the magnitude of which depends on the operating temperature [46].

(2) $$\frac{\Delta S}{n}=-18.449-0.01283\cdot T$$

### 2.2. Irreversible Overpotential

As a result of the polarization phenomenon during the electrochemical reaction of the HT-PEMFC, the actual output potential is lower than the ideal reversible potential. Polarization phenomena typically result in three types of polarization overpotentials: activation overpotential $E_{act}$, concentration overpotential $E_{con}$, and ohmic overpotential $E_{ohm}$. In fact, the leakage current also has an effect on the concentration overpotential and the activation overpotential. Thus, it is not possible to ignore the losses caused by leakage currents.

Activation overpotential [38]:

(3) $$E_{act}=\frac{RT}{\alpha nF}ln(\frac{I+I_{leak}}{I_{0}})\;$$

(4) $$lnI_{leak}=\left(-2342.9\frac{1}{T}+9.0877\right)$$

where α is the charge transfer coefficient, α=0.25 [39]; I is the operating current density; $I_{leak}$ is the leakage current density; and $I_{0}$ is the exchange current density.

Concentration overpotential:

(5) $$E_{con}=\left(1+\frac{1}{\alpha }\right)\frac{RT}{nF}ln(\frac{I_{L}}{I_{L}-I-I_{leak}})$$

where $I_{L}$ is the limiting current density, $I_{L}=2$ (A/cm^2^) [44].

Ohmic overpotential [25]:

(6) $$E_{ohm}=I\left(\frac{l_{m}}{\sigma_{mem}}\right)$$

where $l_{m}$ is the thickness of the PEM, and $\sigma_{mem}$ is the proton conductivity of the PEM electrolyte [47].

(7) $$\sigma_{mem}=\frac{ab}{T}e^{\frac{-c_{act}}{RT}}$$

where a, b are two factors. $c_{act}$ is the activation energy. a, b, and $c_{act}$ are fitted from experimental data [44].

(8) $$a=68DL^{3}-6324DL^{2}+65750DL+8460$$

(9) $$b=\{\begin{array}{c} 1+\left(0.01704T-4.767\right)RH\;373.15K\leq T\leq 413.15\\ 1+\left(0.1432T-56.89\right)RH\;\;413.15K<T\leq 453.15\\ 1+\left(0.7T-309.2\right)RH\;\;\;\;\;\;\;\;\;453.15<T\leq 473.15 \end{array}$$

(10) $$c_{act}=-619.6DL+21750$$

where DL is the phosphoric acid doping level. The doping level, depending on the phosphoric acid concentration, doping temperature, and soaking time [48], is defined as the number of phosphoric acid molecules per polybenzimidazole [25]. RH is the relative humidity of the electrolyte.

### 2.3. Power Density and Efficiency of HT-PEMFC

The actual output voltage $E_{cell}$:

(11) $$E_{cell}=E_{r}-E_{con}-E_{act}-E_{ohm}$$

The output power density P:

(12) $$P=E_{cell}\cdot I=(E_{r}-E_{act}-E_{ohm}-E_{con})\cdot I$$

The output efficiency η:

(13) $$\eta =\frac{P_{f}}{-\Delta \dot{H}}=-\frac{nF\times (E_{r}-E_{act}-E_{ohm}-E_{con})}{\Delta h\left(T\right)}$$

where $-\Delta \dot{H}=-\frac{IA_{0}\Delta h}{nF}$ is the total energy released per unit time [49]; $A_{0}$ is the effective reaction area of a single PEMFC, $A_{0}=0.06$ m^2^; $P_{f}=PA_{0}$ is output power; and Δh(T) is the molar enthalpy change of the electrochemical reaction. Since the efficiency value is always less than 1, which is too small compared to the power density value, the dimensionless power $P^{*}=P/10$ is used for analysis in this paper for clearer comparison and optimization [50].

### 2.4. Model Verification

**Figure 1:**
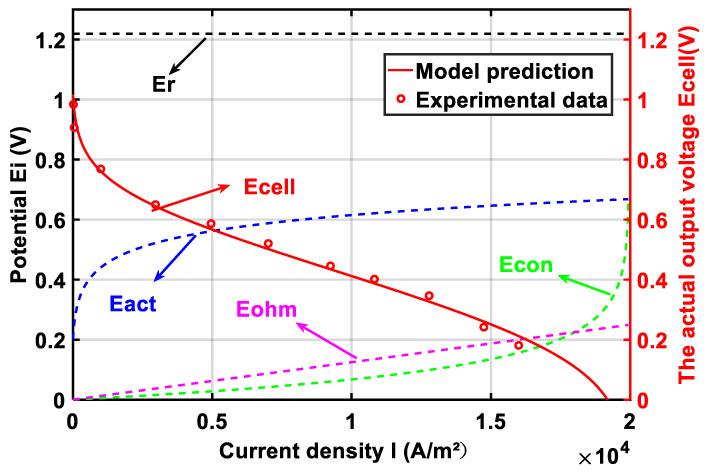
Comparisons of the HT-PEMFC output voltage between the modeling results and the experimental data.

Figure 1 is a comparison of model prediction and experimental data from Ref. [51] under operating temperature T=448 K, where $p_{H_{2}}=1$ atm, $p_{O_{2}}=1$ atm, RH=3.8%; $l_{m}=0.005$ cm, and DL=10. The results show that the model is in good agreement with the experimental data.

## 3. Parametric Studies and Optimization

### 3.1. Effect of Operating Parameters

**Figure 2:**
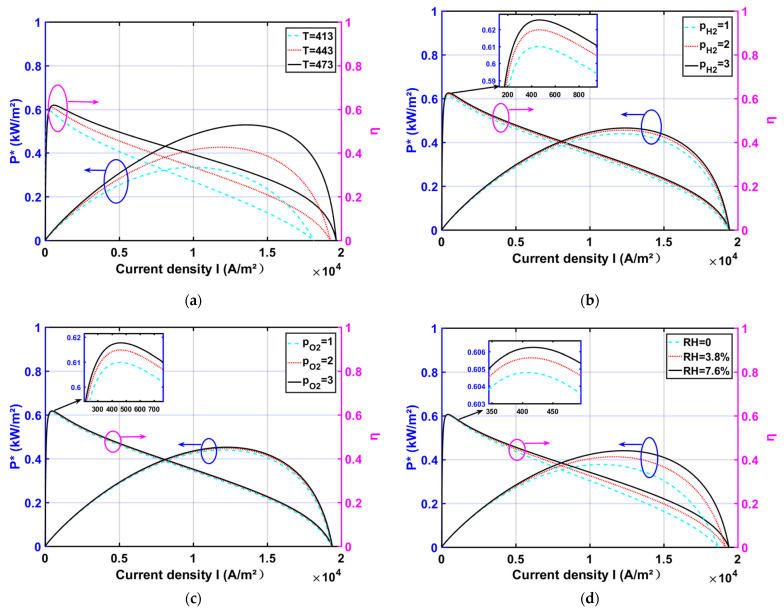
The effect of operating parameters on power density and efficiency (T=448 K; $p_{H_{2}}=1$ atm; $p_{O_{2}}=1$ atm; RH=0.038; $l_{m}=0.005$ cm; DL=10 ): (**a**) different T; (**b**) different $p_{H_{2}}$; (**c**) different $p_{O_{2}}$; (**d**) different RH.

When the HT-PEMFC design parameters are determined, the output performance of the HT-PEMFC is related to T, $p_{H_{2}}$, $p_{O_{2}}$, and RH. The impact of operating parameters on the output performance of the HT-PEMFC is shown in Figure 2.

As shown in Figure 2a, $P^{*}$ and η increase as the operating temperature rises. From the electrochemical kinetics point of view, the increase in temperature facilitates the improvement of proton conductivity and largely reduces the irreversible effects brought by ohmic polarization. From the point of view of molecular dynamics, the conditions of increased operating temperature are favorable to increase the proton reaction rate and shorten the proton transport time, thus improving the output performance of the HT-PEMFC.

As shown in Figure 2b,c, $P^{*}$ and η are increased slightly with increasing inlet gas pressure $p_{H_{2}}$ and $p_{O_{2}}$. The increase in inlet pressure is conducive to the increase in gas diffusion rate in both poles of the battery, and at the same time, it helps the water vapor in the membrane discharge, improving the battery water management. In addition, the difference in gas concentration between the two poles decreases with the increase in air pressure, thus reducing the concentration difference polarization. It can also be found from Equation (1) that $E_{r}$ increases with the increase in $p_{H_{2}}$ and $p_{O_{2}}$, resulting in the increase in the actual output voltage $E_{cell}$.

Figure 2d shows that $P^{*}$ and η both increase with the increase in RH. Since increasing RH facilitates an increase in proton conductivity, it reduces the obstruction of protons through the high-temperature membrane and improves mass transfer, leading to a decrease in ohmic overpotential. The increase in $P^{*}$ and η is more obvious in the high-current density region. In the low-current density region, the ohmic overpotential is relatively low. While in the high-current density region, the ohmic loss increases, and the gain effect from the increasing *RH* is obvious.

### 3.2. Effect of Design Parameters

High-temperature PEM requires good proton conduction ability, stable electrochemical performance, and good toughness to facilitate assembly. Phosphoric acid doping RH and film thickness $l_{m}$ have a great influence on the output performance of the HT-PEMFC. Phosphoric acid doping is the ratio of the quality difference after acidification and before acidification to the quality before acidification.

**Figure 3:**
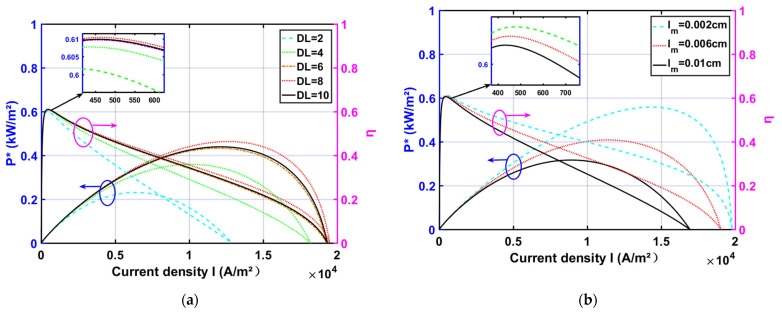
The effect of design parameters on power density and efficiency (T=448 K; $p_{H_{2}}=1$ atm; $p_{O_{2}}=1$ atm; RH=0.038; $l_{m}=0.005$ cm; DL=10 ): (**a**) different DL; (**b**) different $l_{m}$.

Figure 3a shows that both $P^{*}$ and η improve and then decrease as RH increases, reaching a maximum when RH is 8. A certain degree of RH increase is beneficial to improving the proton conductivity. The increase in proton conductivity facilitates the reduction of irreversible losses due to ohmic overpotential, which in turn improves $P^{*}$, η. However, too much phosphoric acid doping will damage the structure of PBI, which will then affect the attachment rate of phosphoric acid and lead to a decrease in proton conductivity.

Figure 3b shows that both $P^{*}$ and η are elevated as $l_{m}$ decreases; $l_{m}$ is one of the main influencing factors for waste heat generation. The thickening of the high-temperature membrane $l_{m}$ increases the path length of the ions between the anode and cathode, which leads to an increase in the ohmic overpotential of the HT-PEMFC. Therefore, when choosing high-temperature film, thinner film should be selected as much as possible. In the low-current density region, the effect of $l_{m}$ on $P^{*}$ and η is not significant. Since the ohmic overpotential is small at low current density, decreasing $l_{m}$ results in almost no improvement in $P^{*}$, η.

### 3.3. Finite Time Thermodynamic Optimization

Finite time thermodynamic optimization is mainly used to solve the extreme value problem in irreversible processes, and its application in engineering is mainly to explore the coordination between various performance parameters of the system, so as to obtain the optimal performance output and the optimal system structure. The output performance of a single HT-PEMFC is mainly related to the operating parameters (T, $p_{H_{2}}$, $p_{O_{2}}$, RH) and structural parameters ($l_{m}$, DL).

#### 3.3.1. Multi-Objective Optimization Model

PSO has been widely used in parametric optimization problems because of its simplicity and fast convergence [52,53]. The mathematical model using the PSO algorithm is shown in the following Equation (14).

(14) $$\{\begin{array}{c} maxf\left(x\right)\\ x_{i}={\left[x_{1},x_{2},\ldots ,x_{l}\right]}^{T},i=1,2,\ldots ,l\\ s.t.\;\;g_{j}\left(x\right)\geq 0,j=1,2\ldots ,m \end{array}$$

where f(x) is the objective function; x is the optimization variable; $x_{n}$ is the parameter to be optimized; l is the number of variables to be optimized; $g_{j}\left(x\right)$ is the inequality constraint; and m is the number of constraints.

The optimized objective function is shown in Equation (15) below. The constraint is $0\leq I=g_{1}\left(x\right)\leq 20000$.

(15) $$f\left(x\right)=\omega_{1}f_{1}\left(x\right)+\omega_{2}f_{2}\left(x\right)$$

In the optimization model, $f_{1}=P^{*}$, $f_{2}=\eta$. The use of dimensionless power ensures that the evaluation function 0<f<1, and a larger value of f indicates a better optimization effect. $f_{1}\left(x\right)$ and $f_{2}\left(x\right)$ are two different functions of x; the weighting factors $\omega_{1}$ and $\omega_{2}$ are as follows [54]:

(16) $$\{\begin{array}{c} \omega_{1}=\left(f_{2}^{2}-f_{2}^{1}\right)/\left[\left(f_{2}^{2}-f_{2}^{1}\right)+\left(f_{1}^{1}-f_{1}^{2}\right)\right]\\ \omega_{2}=\left(f_{1}^{1}-f_{1}^{2}\right)/\left[\left(f_{2}^{2}-f_{2}^{1}\right)+\left(f_{1}^{1}-f_{1}^{2}\right)\right] \end{array}.$$

Here, $f_{2}^{2}$ and $f_{1}^{1}$ are the maximum values of $f_{2}\left(x\right)$ and $f_{1}\left(x\right)$, respectively, $f_{2}^{1}$ is the value of $f_{2}\left(x\right)$ corresponding to the maximum value of $f_{1}\left(x\right)$, and $f_{1}^{2}$ is the value of $f_{1}\left(x\right)$ corresponding to the maximum value of $f_{2}\left(x\right)$.

**Figure 4:**
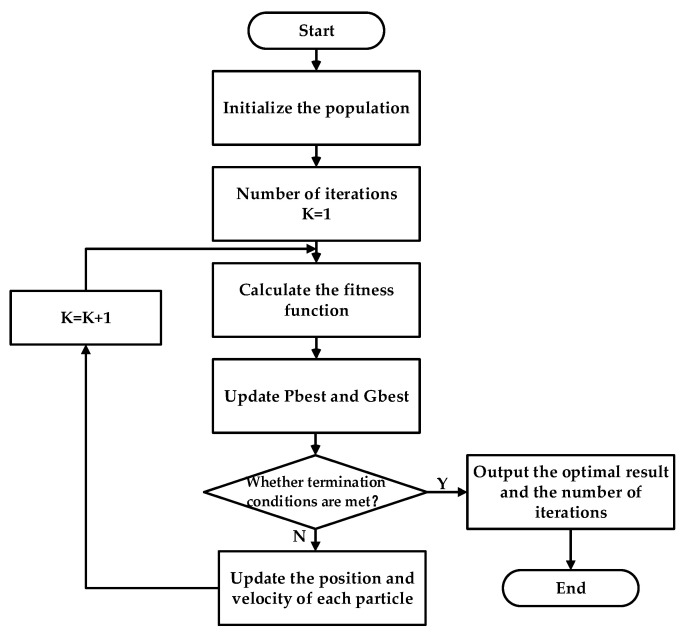
PSO flow chart.

The PSO process is shown in Figure 4; the implementation steps are as follows:

1. Initialize the population: the particle swarm size is 500 and the maximum number of iterations is 500. The maximum flight speed of the particle is 10% of the optimization variables. Initialize the random position and velocity of each particle.
2. Calculate the fitness value: the fitness value of each particle is evaluated by the objective function f(x).
3. Update the particle best value (Pbest) and global best value (Gbest): the particle velocity and position update equation are as shown in Equation (17), where $V_{id}^{k}$ and $X_{id}^{k}$ are the velocity and position of the $i^{th}$ particle after the k iteration, respectively. $r_{1}$ and $r_{2}$ are random numbers between [0,1]. The learning factor $c_{1}=c_{2}=2$ and the inertia weight ω=0.8.
  (17) $$\{\begin{array}{c} V_{id}^{k+1}=\omega V_{id}^{k}+c_{1}r_{1}\left(Pbest_{id}^{k}-X_{id}^{k+1}\right)+c_{2}r_{2}\left(Gbest_{id}^{k}-X_{id}^{k+1}\right)\\ X_{id}^{k+1}=X_{id}^{k}+V_{id}^{k} \end{array}$$
4. Judgment: the termination condition selects the maximum number of iterations. If the condition is satisfied, the optimal solution will be output.

#### 3.3.2. Optimization of Operating and Design Parameters

The optimal parameters of the HT-PEMFC were obtained through the following two steps: first, optimal operating parameters were sought for a given single HT-PEMFC; second, we obtained the optimal design parameters of a single HT-PEMFC under the optimal operating parameters.

When RH and $l_{m}$ are determined, f(x) is a multivariate function with respect to T, $p_{H_{2}}$, $p_{O_{2}}$, and RH. The optimization variables are $x_{4}=\left[T,p_{H_{2}},p_{O_{2}},RH\right]$; the range of optimization variables is 413≤T≤473, $1\leq p_{H_{2}}\leq 3$, $1\leq p_{O_{2}}\leq 3$, and 0≤RH≤7.6%. The output optimization variable is $x_{4}=\left[473,3,3,7.6\%\right]$.

When the single HT-PEMFC is working at the optimal operating parameters, f(x) is related to DL and $l_{m}$. The optimization variables are $x_{2}=\left[DL,lm\right]$, and the range of optimization variables is 2≤DL≤10 and $2\times 10^{-5}\leq l_{m}\leq 1\times 10^{-4}$. The output optimization variable is $x_{2}=\left[8.4,2\times 10^{-5}\right]$.

**Figure 5:**
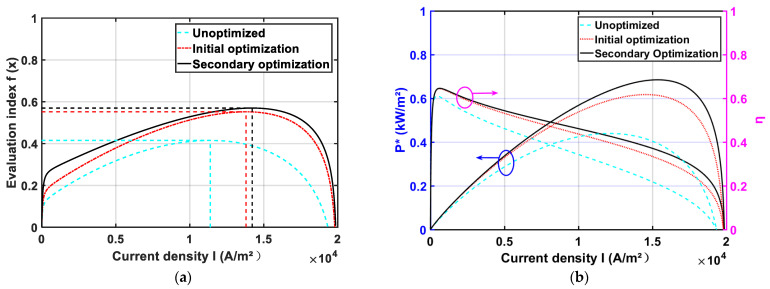
Comparison of optimization results: (**a**) f(x); (**b**) $P^{*}$, and η.

Figure 5 shows the comparison of optimization results. Figure 5a shows that the evaluation function f(x) has increased after the initial optimization and the secondary optimization. Figure 5b demonstrates that both $P^{*}$ and η increase after the optimization, and the performance improvement of the HT-PEMFC is more pronounced by optimizing the operating parameters. The results show that the output performance of a single HT-PEMFC optimized by the PSO algorithm is improved.

**Figure 6:**
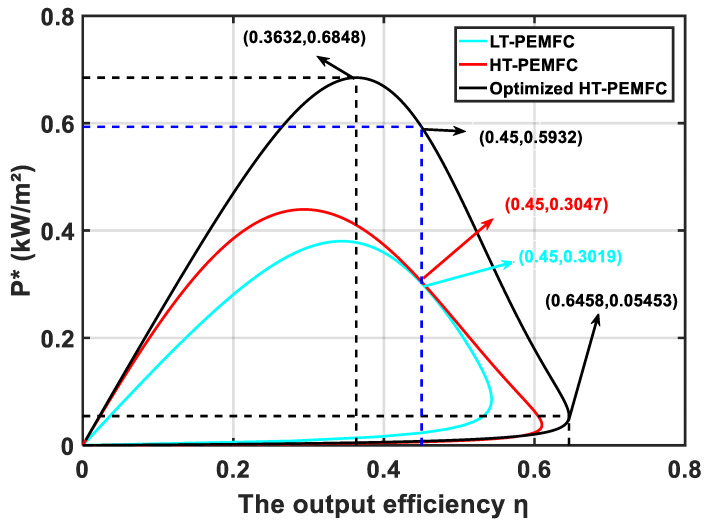
Comparison of $P^{*}$ and η curves of the LT-PEMFC, HT-PEMFC, and optimized HT-PEMFC.

Figure 6 shows the output performance comparison curves of the LT-PEMFC [37], HT-PEMFC, and optimized HT-PEMFC. As shown in Figure 6, the power density and efficiency of the optimized HT-PEMFC are relatively high. When the output efficiency of the optimized HT-PEMFC single cell is 36.32%, the corresponding maximum power density is 6.848 kW/m^2^. When the power density of the optimized HT-PEMFC single cell is 0.5453 kW/m^2^, the corresponding maximum output efficiency is 64.58%.

## 4. FCV Powertrain Design

The powertrain parameters should be matched according to the vehicle parameters and design requirements. The vehicle parameters and design requirements are shown in Table 1.

### 4.1. Configuration

**Figure 7:**
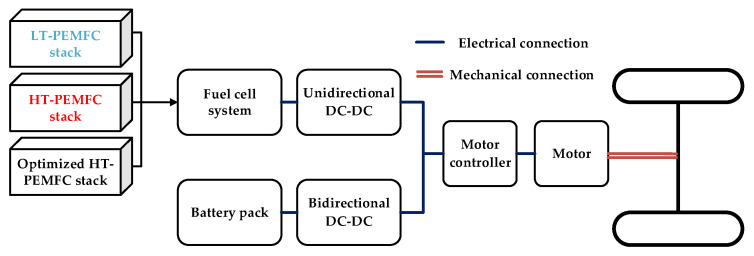
Different PEMFC schemes of the FC + B powertrain.

According to the above PEMFC single-cell output performance, different configuration schemes are available for FCV. Although the pure fuel cell drive (PFC) has a simple structure and is easy to control, it also has obvious disadvantages, such as low power density, slow power response, and inability to recover braking energy. The low specific energy, limited energy storage, short peak power duration, and high matching control requirements with other system components of the ultra-capacitor restrict the development of fuel cell and ultra-capacitor hybrid power systems. Therefore, this paper selects the configuration of fuel cell and battery combined drive (FC + B), which is widely used and representative at present. The FC + B powertrain works more efficiently, has better cold start performance, and can recover some of the braking energy [55]. Since the output voltage of the fuel cell is not quite stable during operation, a DC-DC converter is connected in series on the circuit to ensure that the output voltage can be a constant value when the input voltage fluctuates within its range. Therefore, the powertrain schemes for the FCV are shown in Figure 7.

### 4.2. Motor Parameters

#### 4.2.1. Maximum Power and Rated Power

The power demand to meet the maximum speed is $P_{max1}$. The power demand to meet the maximum climbing degree is $P_{max2}$. The power demand to meet the acceleration time is $P_{max3}$. $P_{max1}$,$\;P_{max2}$, and $P_{max3}$ are shown in Equation (18) [56,57].

(18) $$\{\begin{array}{c} P_{max1}=\frac{u_{max}}{3600\eta_{t}}\left(mgf+\frac{C_{D}Au_{max}^{2}}{21.15}\right)\\ P_{max2}=\frac{u_{p}}{3600\eta_{t}}\left(mgf\cos\alpha_{max}+mg\sin\alpha_{max}+\frac{C_{D}Au_{p}^{2}}{21.15}\right)\\ P_{max3}=\frac{1}{3600\eta_{t}}\left(mgf\frac{u_{e}}{1.5}+\frac{C_{D}Au_{e}^{3}}{52.875}+\delta m\frac{u_{e}^{2}}{7.2t_{e}}\right) \end{array}$$

where $u_{p}$ is the speed of climbing, which is 30 km/h; $\alpha_{max}$ is the maximum slope angle, where $\alpha_{max}=\arctan\left(i_{max}\right)$; $u_{e}$ is the velocity at acceleration termination, which is 100 km/h; $\eta_{t}=0.9$ is the efficiency of the powertrain; and δ=1.05 is the rotation mass conversion factor. The maximum power of the motor $P_{emax}$ should meet the requirements of maximum speed, maximum climbing, and acceleration time at the same time, as shown in Equation (19).

(19) $$P_{emax}\geq \max\left(P_{max1}\;P_{max2}\;P_{max3}\right)$$

Considering that the FCV should have a certain backup power when accelerating or climbing, 100 kW was considered as the motor maximum power $P_{emax}$. Since the rated power of the motor $P_{e}$ needs to meet the power requirements at the maximum speed [58], $P_{e}$ is chosen to be 55 km/h.

#### 4.2.2. Maximum Speed and Rated Speed

The maximum speed of the motor $n_{max}$ is determined by $u_{max}$ and the transmission ratio, as shown in Equation (20).

(20) $$n_{max}=\frac{u_{max}i_{o}i_{g}}{0.377r}$$

where $i_{o}$ is the final drive ratio ($i_{o}=1$) and $i_{g}$ is the gearbox ratio. Since the motor has good working characteristics and speed regulation characteristics, only a relatively small number of gears are required to meet the design requirements. The one-speed gearbox was selected with the gearbox ratio $i_{g}=8$ through reviewing extensive literature. In order to ensure that the motor speed has a certain amount of redundancy, the maximum motor speed $n_{max}$ is 10,000 r/min. The rated speed of the motor $n_{e}$ is as shown in Equation (21) below.

(21) $$n_{e}=\frac{n_{max}}{\beta }$$

where β=2.5 is the motor expansion constant power area factor. Thus, 4000 r/min is taken as the motor rated speed.

#### 4.2.3. Maximum Torque and Rated Torque

The motor rated torque $T_{e}$ is shown as Equation (22).

(22) $$T_{e}=\frac{9550P_{e}}{n_{e}}$$

The motor maximum torque $T_{max}$ is shown as Equation (23).

(23) $$T_{max}=\lambda T_{e}$$

where λ=2.7 is the overload factor. By the calculation, the maximum torque is selected as 360 Nm, and the rated torque is selected as 131.31 Nm.

### 4.3. Fuel Cell Parameters

The fuel cell system should be able to meet the maximum speed requirements and maintain the operation of ancillary equipment. Considering the efficiency of the system, the fuel cell system power $P_{fc}$ is as shown in Equation (24).

(24) $$P_{fc}=\frac{P_{e}}{\eta_{DC}\eta_{mc}}+P_{acc}$$

where $\eta_{inv}=0.9$ is the efficiency of the motor inverter; $\eta_{DC}=0.9$ is the efficiency of the DC/DC converter; $P_{acc}=5\;\mathrm{kW}$ is the power required by the automobile auxiliary equipment. In consideration of certain redundancy,$\;P_{fc}$ is 75 kW.

Based on the above model, the power density and efficiency variation curves of the PEMFC single cell are shown in Figure 6. Since the maximum efficiency of the fuel cell passenger vehicle needs to be greater than or equal to 45%, the power density at 45% efficiency is chosen as the maximum power density. Thus, the number of PEMFC single cells N can be determined by Equation (25).

(25) $$N=\frac{P_{fc}}{P_{f}}=\frac{P_{fc}}{P\times A_{0}}$$

$N_{1}=415$ is the single cell number of the LT-PEMFC stack; $N_{2}=411$ is the single cell number of the HT-PEMFC stack; and $N_{3}=211$ is the single cell number of the optimized HT-PEMFC stack. Since the optimized HT-PEMFC single cell has a higher power density, the number of PEMFC single cells is less, which is more conducive to the vehicle’s spatial layout and light weight.

### 4.4. Battery Parameters

Considering the system efficiency, the battery power $p_{b}$ is shown in Equation (26).

(26) $$P_{b}=\frac{P_{emax}}{\eta_{DC}\eta_{inv}}-P_{fc}+P_{acc}$$

In order to ensure a certain redundancy of the battery, $P_{b}$ is selected as 55 kW. The battery type in this paper is lithium-ion battery.

Finally, the parameters of the fuel cell powertrain are briefly described as shown in Table 2.

## 5. Results and Discussions

A secondary development for the Advanced Vehicle Simulator (ADVISOR) is applied to develop matched FCV models, and the power and efficiency curves of different types of PEMFCs are imported. FCV uses different types of PEMFC stacks for simulation testing [59]. The results of the dynamic test are $u_{max}=183.9$ (km/h), $i_{max}=38.5\%$, and $t_{e}=9.2$ s. Since the total power of the three types of PEMFC stacks is the same, the dynamics test results under different driving cycles should be the same without considering the weight of the PEMFC stacks. The simulation results show that the dynamic performance meets the design requirements. Since the hydrogen consumption of the FCV is different under different driving cycles, this paper selects four driving cycles: the Chinese Typical City Driving Cycle (CCDC), New Europe Driving Cycle (NEDC), Urban Dynamometer Driving Schedule (UDDS) [60], and Highway Fuel Economy Test Cycle (HWTFE), as the test conditions for simulation and comparison.

**Figure 8:**
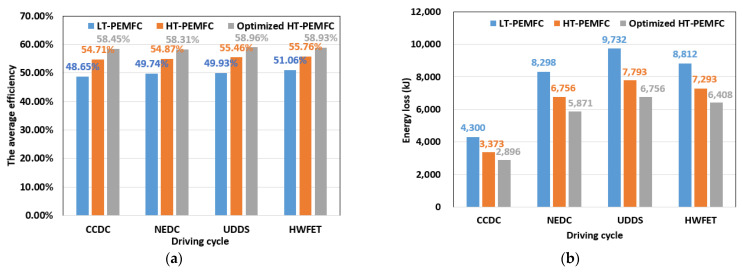
Comparison of fuel cell system efficiency and energy loss: (**a**) Efficiency; (**b**) Energy loss.

Figure 8 shows a comparison of the fuel cell system efficiency and energy loss. From Figure 8a, the optimized HT-PEMFC has the highest average efficiency under all four driving cycles, which can reach about 58%. From Figure 8b, the HT-PEMFC has the lowest energy loss in all four driving cycles. Therefore, the average efficiency of the fuel cell system can be improved, and the energy loss can be reduced by optimizing the operating and design parameters of the HT-PEMFC.

**Figure 9:**
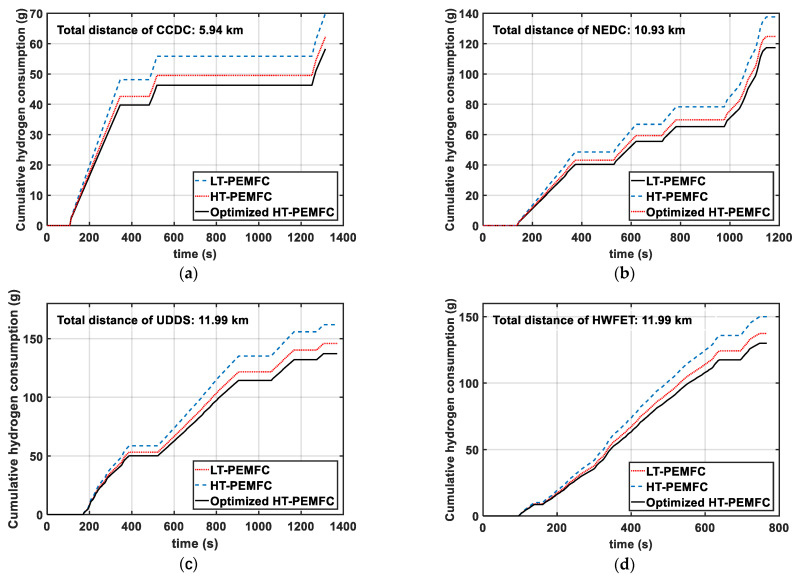
Comparison of cumulative hydrogen consumption under different driving cycles: (**a**) CCDC; (**b**) NEDC; (**c**) UDDS; (**d**) HWFET.

Figure 9 shows the comparison of the hydrogen consumption under four driving cycles. From Figure 9 it can be seen that the optimized HT-PEMFC has lower hydrogen consumption. As a result of the higher output efficiency of the optimized HT-PEMFC, there are fewer irreversible losses, which leads to lower hydrogen consumption. From Table 3 below, it can also be obtained that the 100 km hydrogen consumption of the optimized HT-PEMFC stack is the lowest. Therefore, the application of the optimized HT-PEMFC in FCVs is beneficial for improving vehicle economy.

## 6. Conclusions

In this paper, a thermodynamic model of a single HT-PEMFC using phosphoric acid-doped PBI film is developed, in which the irreversible effects of various polarization losses and leakage currents are considered. Based on the analysis and optimization of the thermodynamic performance of the HT-PEMFC single cell, the FCV power system was designed, and the performance of the FCV under different operating conditions was analyzed. The main conclusions are as follows:

- The reliability of the model was verified by comparing the HT-PEMFC model with the experimental data. By the parametric studies, the appropriate increase in T, $p_{H_{2}}$, $p_{O_{2}}$, and RH is beneficial to the HT-PEMFC output performance improvement. With increasing the doping level DL, the output performance increases and then decreases. With the decrease in proton film thickness $l_{m}$, the output performance is improved;
- The PSO algorithm can optimize the power density and efficiency of the HT-PEMFC single cell based on finite-time thermodynamic theory. The simulation results show that the performance of the optimized HT-PEMFC single cell is improved, the power density can be obtained up to 6.848 kW/m^2^, and the efficiency can reach up to 64.58%;
- Three different powertrain solutions are available for FCVs based on the different power density and efficiency curves of the LT-PEMFC, HT-PEMFC, and optimized HT-PEMFC outputs. The simulation comparison shows that the optimized HT-PEMFC stack has the lowest number of single cells, which is conducive to the vehicle’s structural arrangement and light weight. Moreover, the FCV that applied the optimized HT-PEMFC has the highest average efficiency, the lowest energy loss, and the lowest 100 km hydrogen consumption.

The derived conclusion may provide some directions and references for future research related to the influence of parameters on HT-PEMFC performance, improvement methods of HT-PEMFC output performance, and the design of FCV powertrain.

## Figures and Tables

**Table 1:** Vehicle parameters and design requirements.

Vehicle Parameters	Value	Design Requirements	Value
Mass m (kg)	1850	Maximum speed $u_{max}$ (km/h)	150
Rolling resistance coefficient f	0.012	0–100 km/h acceleration time $t_{e}$ (s)	10
Air resistance coefficient $C_{D}$	0.32	Maximum climb at 30 km/h $\;i_{max}$ (%)	30
Windward area A(m^2^)	2.4		
Wheel rolling radius r(m)	0.33		

**Table 2:** The parameters of the FCV powertrain.

Powertrain Components	Parameters	Values
Motor	$P_{e}\left(P_{emax}\right)$ (kW)	55 (100)
	$n_{e}$ ($n_{max}$) (r/min)	4000 (1000)
	$T_{e}$($T_{max}$) (Nm)	132 (360)
Fuel cell	$P_{fc}$ (kW)	75
	Type	LT-PEMFC; HT-PEMFC; Optimized HT-PEMFC
Battery	$P_{b}$/kW	55
	Type	Lithium-ion

**Table 3:** 100 km hydrogen consumption (g) under different driving cycles.

Driving Cycles	LT-PEMFC	HT-PEMFC	Optimized HT-PEMFC
CCDC	1178.19	1047.78	980.65
NEDC	1259.08	1141.45	1073.99
UDDS	1351.05	1216.25	1144.13
HWFET	908.91	832.23	787.53

## Data Availability

Not applicable.
